# Electroacupuncture Alleviates Neuropathic Pain by Inhibiting Spinal CCL2-Driven Microglial Activation

**DOI:** 10.3390/ijms26189049

**Published:** 2025-09-17

**Authors:** Vishnumolakala Sindhuri, Min-Jae Koo, Seung Heon Jeon, Ki-Tae Ha, Seungtae Kim, Sungtae Koo

**Affiliations:** 1 Research Institute for Korean Medicine, Pusan National University, Yangsan 50612, Republic of Korea; sindhu17@pusan.ac.kr (V.S.); 67ggs@naver.com (M.-J.K.); 2 Department of Korean Medical Science, School of Korean Medicine, Pusan National University, Yangsan 50612, Republic of Korea; zegehio@naver.com (S.H.J.); hagis@pusan.ac.kr (K.-T.H.); kimst@pusan.ac.kr (S.K.); 3 Department of Korean Medicine, School of Korean Medicine, Pusan National University, Yangsan 50612, Republic of Korea

**Keywords:** acupuncture, chronic pain, chemokine receptor, inflammatory cytokines, microglia

## Abstract

Electroacupuncture (EA) has shown analgesic potential for neuropathic pain, yet its underlying molecular mechanisms remain incompletely understood. This study aimed to investigate whether EA relieves neuropathic pain by modulating CCL2/CCR2 signaling and microglial activation in the spinal cord. Neuropathic pain was induced in rats by L5 spinal nerve ligation. EA was administered at acupoints ST36 and GB34 (1 mA, 2 Hz, 30 min) daily from postoperative days 3 to 7. Rats were assigned to anesthetized control (ANE), non-acupoint stimulation (NAP), and acupoint stimulation (ACU) groups. Pain behavior was evaluated using paw withdrawal threshold and latency. Western blot and immunofluorescence were used to assess CCL2, CCR2, Iba1, IL-1β, and TNF-α expression in the L4–L6 spinal cord. EA significantly attenuated mechanical allodynia and thermal hyperalgesia in the ACU group, accompanied by reductions in CCL2, CCR2, microglial marker Iba1, and pro-inflammatory cytokines. Most importantly, intrathecal administration of recombinant CCL2 completely abolished EA’s analgesic effects, establishing the causal necessity of CCL2/CCR2 signaling in EA-mediated analgesia. These findings suggest that EA exerts its analgesic effects through downregulation of the CCL2/CCR2 pathway and inhibition of microglial activation. The reversal of EA’s effects by exogenous CCL2 supports the critical role of spinal chemokine signaling in EA-mediated analgesia.

## 1. Introduction

Neuropathic pain represents one of the most difficult clinical conditions in modern medicine, affecting approximately 7–10% of the global population and significantly impairing quality of life [1,2]. This condition arises from somatosensory nervous system injury or dysfunction, characterized by spontaneous pain, hyperalgesia, and allodynia that persist beyond tissue healing [3]. The pathophysiology of neuropathic pain involves complex interactions between injured neurons and non-neuronal cells, with spinal microglia emerging as critical mediators in both the initiation and maintenance of chronic pain states [4,5,6]. Following peripheral nerve injury, spinal microglia undergo rapid morphological and functional transformation from a resting ramified state to an activated amoeboid phenotype, releasing a cascade of pro-inflammatory mediators that amplify nociceptive signaling [7,8]. Among the numerous signaling molecules involved in microglial activation, chemokine networks have gained particular attention, with the CCL2 (C-C motif chemokine ligand 2) and its cognate receptor CCR2 representing a pivotal axis in neuropathic pain development [9,10]. The CCL2/CCR2 signaling pathway not only facilitates the recruitment and activation of both resident and infiltrating microglia but also orchestrates sustained neuroinflammatory responses that underlie the chronification of pain [11,12]. Importantly, genetic ablation or pharmacological blockade of CCR2 has been consistently shown to attenuate neuropathic pain behaviors in preclinical models, establishing this chemokine axis as a promising therapeutic target [13].

Electroacupuncture (EA), a modernized form of traditional acupuncture that combines needle insertion with controlled electrical stimulation, has emerged as a promising therapeutic strategy for managing various chronic pain conditions, including neuropathic pain [14,15]. Clinical studies have demonstrated EA’s efficacy in reducing pain intensity and improving functional outcomes in patients with diabetic neuropathy, postherpetic neuralgia, and chemotherapy-induced peripheral neuropathy [16,17]. The analgesic mechanisms of EA are multifaceted, involving the modulation of neurotransmitter systems, activation of endogenous opioid pathways, and importantly, the suppression of neuroinflammatory processes [18,19]. Recent preclinical investigations have provided compelling evidence that EA can effectively reduce spinal microglial activation and attenuate the production of pro-inflammatory cytokines such as interleukin-1β (IL-1β), tumor necrosis factor-α (TNF-α), and interleukin-6 (IL-6) in animal models of neuropathic pain [20,21]. Furthermore, EA treatment has been shown to modulate glial cell morphology, reduce the expression of microglial activation markers, and restore the balance between pro-inflammatory and anti-inflammatory mediators in the spinal cord [22,23]. However, despite these promising findings demonstrating EA’s anti-inflammatory and glial-modulating effects, the specific impact of EA on the CCL2/CCR2 signaling pathway—a critical driver of microglial activation in neuropathic pain—has not been fully elucidated, representing a significant gap in our mechanistic understanding of EA’s therapeutic actions.

While existing studies have provided valuable insights into EA’s effects on neuroinflammation and microglial function, most investigations have demonstrated that EA treatment is associated with reduced inflammatory markers, but could not establish whether these changes represent a direct causal mechanism of analgesia or merely secondary consequences of pain relief [24]. These correlational findings, though informative, cannot definitively determine whether CCL2/CCR2 pathway modulation is a cause or consequence of EA’s therapeutic effects. To advance our mechanistic understanding and establish causality, experimental approaches that directly manipulate the proposed target pathway are essential [25]. The limitations of purely correlational studies become particularly evident when considering the complex interactions between pain, inflammation, and neural plasticity. In these systems, multiple interconnected pathways may be simultaneously affected by therapeutic interventions. Therefore, there is a critical need for experimental designs that can isolate and test the specific contribution of individual signaling pathways to EA’s analgesic effects. Intrathecal injection of recombinant CCL2 provides a powerful pharmacological tool to address this limitation. This approach artificially activates the CCL2/CCR2 pathway and examines whether such activation can counteract or reverse EA-induced analgesia [10,26]. This direct manipulation allows researchers to move beyond observational correlations to determine the causal necessity of CCL2/CCR2 signaling in maintaining EA’s therapeutic benefits.

To establish causality rather than mere association, this study employed direct pharmacological manipulation using intrathecal administration of recombinant CCL2 to test whether reactivation of this pathway could reverse EA-induced analgesia. The primary aim was to investigate the causal role of spinal CCL2/CCR2 signaling in electroacupuncture-induced analgesia in a rat model of neuropathic pain and to elucidate the associated changes in microglial activation and cytokine expression. We hypothesized that EA treatment would significantly attenuate neuropathic pain through the downregulation of spinal CCL2 release and subsequent reduction in microglial activation and pro-inflammatory cytokine production. Most importantly, we predicted that intrathecal administration of exogenous recombinant CCL2 would completely reverse EA’s analgesic effects by reactivating the CCL2/CCR2 signaling pathway, thereby providing the first direct causal evidence for the necessity of this chemokine axis in EA-mediated pain relief and advancing our understanding from correlative associations to mechanistic certainty.

## 2. Results

### 2.1. Electroacupuncture Alleviates Mechanical and Thermal Hypersensitivity in Neuropathic Pain

**Figure 1:**
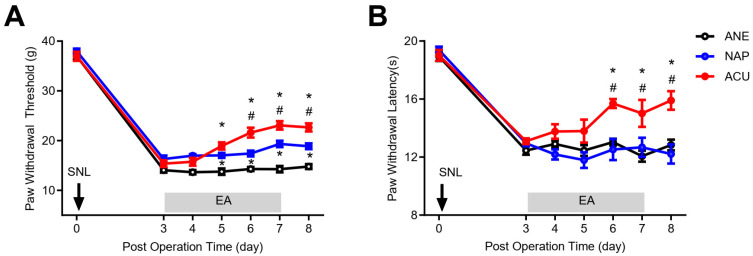
Electroacupuncture (EA) alleviates mechanical allodynia and thermal hyperalgesia following spinal nerve ligation (SNL). (**A**) Paw withdrawal threshold (PWT) was measured by a Dynamic Plantar Aesthesiometer. (**B**) Paw withdrawal latency (PWL) measured with a plantar test apparatus. Both PWT and PWL were significantly improved in the EA-treated (ACU) group compared with the anesthetized control (ANE) and non-acupoint stimulation (NAP) groups. NAP stimulation modestly increased PWT but did not affect PWL. Data are presented as mean ± SEM (*n* = 9 per group). * *p* < 0.05 vs. ANE; # *p* < 0.05 vs. NAP.

Following spinal nerve ligation (SNL), rats developed robust mechanical allodynia and thermal hyperalgesia, as reflected by marked reductions in paw withdrawal threshold (PWT) and paw withdrawal latency (PWL). In the anesthetized control (ANE) group, these pain behaviors persisted throughout the observation period. In contrast, electroacupuncture (EA) at ST36 and GB34 (ACU group) significantly increased PWT and prolonged PWL from postoperative day 5 or 6 through day 8, compared with both ANE and non-acupoint stimulation (NAP) groups (Figure 1A,B). Notably, NAP stimulation produced a modest increase in PWT but failed to improve PWL, highlighting that the analgesic effects were specific to stimulation at acupoints. These findings confirm the point-specific efficacy of EA in alleviating SNL-induced neuropathic pain.

### 2.2. Electroacupuncture Reduces CCL2 Expression and Immunoreactivity in Neuropathic Pain

**Figure 2:**
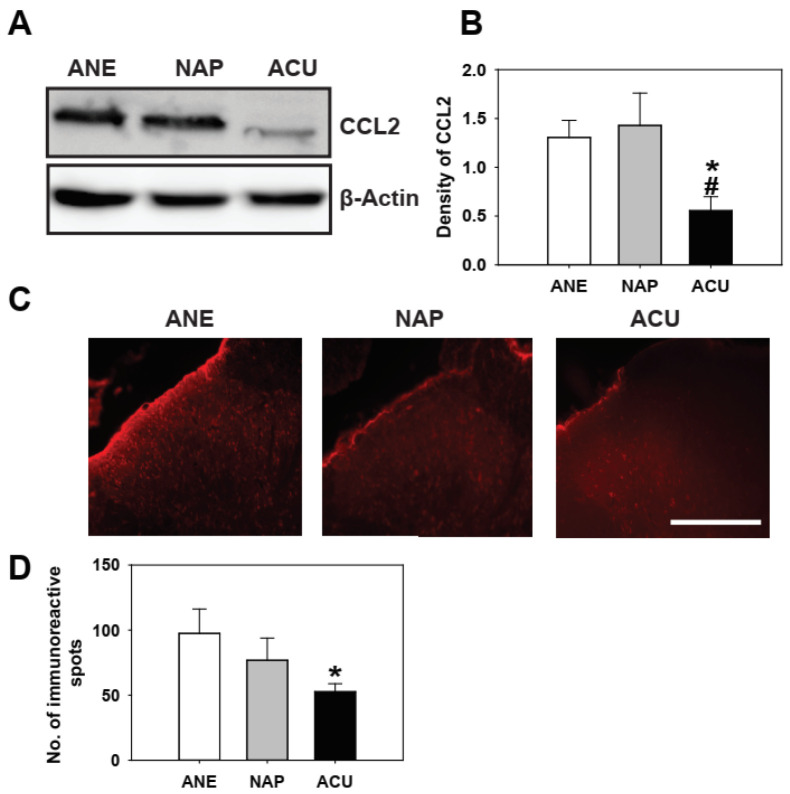
Electroacupuncture (EA) suppresses CCL2 expression in the spinal dorsal horn of rats with neuropathic pain. (**A**) Representative Western blot images of CCL2 protein expression in the L4–L6 spinal dorsal horn from anesthetized control (ANE), non-acupoint stimulation (NAP), and electroacupuncture (ACU) groups. (**B**) Quantification of band density shows that CCL2 protein levels were significantly reduced in the ACU group compared with ANE and NAP groups. (**C**) Representative immunofluorescence images of CCL2 expression in the spinal dorsal horn for each group. Scale bar = 100 μm. (**D**) Quantification of CCL2-positive spots confirms that EA markedly reduces CCL2 expression relative to ANE group. Data are presented as mean ± SEM (*n* = 5 per group for Western blot, *n* = 4 per group for immunofluorescence). * *p* < 0.05 vs. ANE; # *p* < 0.05 vs. NAP.

Western blot and immunofluorescence analyses confirmed that SNL markedly increased CCL2 expression in the spinal dorsal horn compared with naïve controls (Supplementary Figure S1). To determine whether EA could counteract this SNL-induced upregulation, we compared CCL2 levels among experimental groups. Representative immunofluorescence images and quantification (Figure 2C,D) showed that CCL2-positive cells were significantly reduced in the ACU group compared with the ANE and NAP groups. Consistently, Western blot and densitometric analysis (Figure 2A,B) revealed that EA markedly suppressed CCL2 protein expression. These findings indicate that EA effectively attenuates the SNL-induced increase in CCL2 expression in a point-specific manner.

### 2.3. Electroacupuncture Suppresses CCR2 Expression and Reduces CCR2/Iba-1 Co-Localization in Neuropathic Pain

SNL also enhanced CCR2 expression and its association with microglia in the spinal dorsal horn. Western blot and immunofluorescence analyses confirmed that CCR2 levels were significantly elevated in SNL rats compared with naïve controls, with a marked increase in CCR2/Iba-1 co-localization (Supplementary Figure S2). These findings demonstrate that the CCL2/CCR2 axis is robustly activated in microglial populations following peripheral nerve injury.

**Figure 3:**
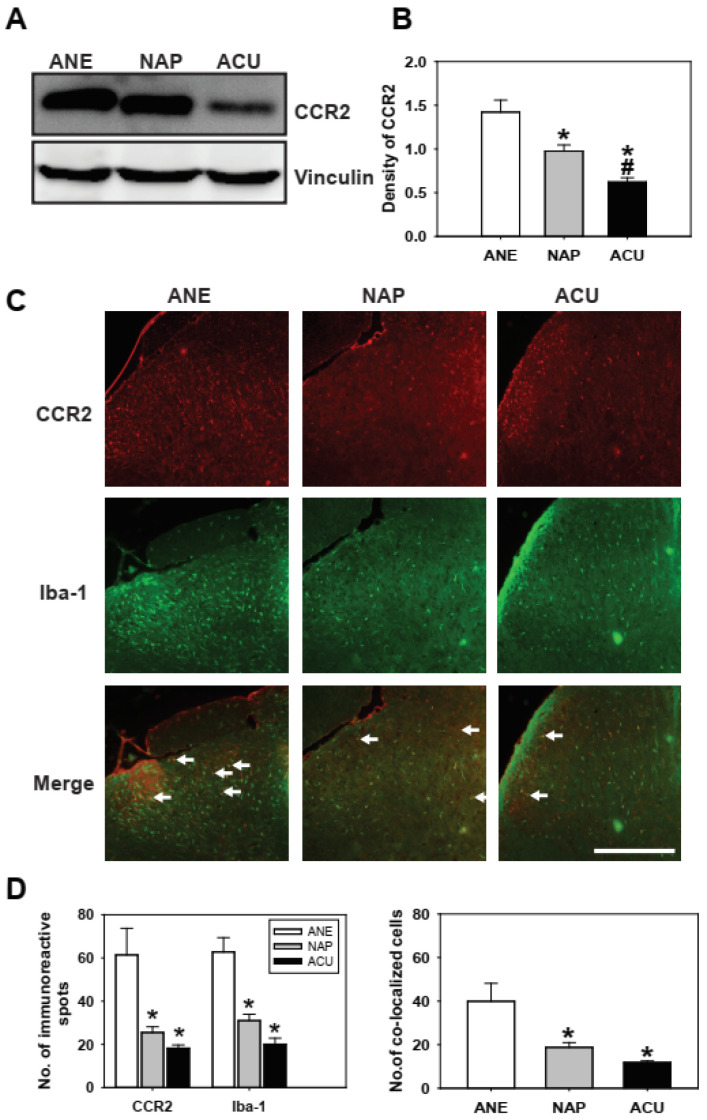
Electroacupuncture (EA) reduces CCR2 expression and CCR2-positive microglial activation in the spinal dorsal horn. (**A**) Representative Western blot image and (**B**) densitometric quantification of CCR2 protein levels in the L4–L6 spinal dorsal horn from the anesthetized control (ANE), non-acupoint stimulation (NAP), and EA (ACU) groups. EA significantly reduced CCR2 expression compared with ANE and NAP. (**C**) Representative immunofluorescence images showing CCR2 (red) and the microglial marker Iba-1 (green). Merged images reveal CCR2/Iba-1 co-localized cells (arrows), which were markedly reduced in the ACU group. Scale bar = 100 μm. (**D**) Quantification of CCR2- and Iba-1-positive immunoreactive cells (left) and CCR2/Iba-1 co-localized cells (right), demonstrating that EA decreased microglial activation compared with ANE. Data are presented as mean ± SEM (*n* = 5 per group for Western blot; *n* = 4 per group for immunofluorescence). * *p* < 0.05 vs. ANE; # *p* < 0.05 vs. NAP.

EA significantly attenuated this activation. As shown in Figure 3, EA at ST36 and GB34 (ACU group) significantly decreased CCR2 protein expression compared with the ANE and NAP groups. Immunofluorescence analysis further revealed that EA reduced the number of CCR2- and Iba-1-positive cells, as well as CCR2/Iba-1 co-localized microglia, relative to both control groups. To further validate these findings, we performed high-magnification imaging of CCR2/Iba-1 immunofluorescence. As shown in Supplementary Figure S3, CCR2 intensity and CCR2/Iba-1 co-localization were markedly increased in the ANE and NAP groups, whereas EA treatment substantially reduced both CCR2 immunoreactivity and its overlap with microglia. These higher-resolution data corroborate the quantitative analyses presented in Figure 3, confirming that EA effectively suppresses CCR2-driven microglial activation in the spinal dorsal horn.

### 2.4. Electroacupuncture Reduces IL-1β and TNF-α Expression in Neuropathic Pain

**Figure 4:**
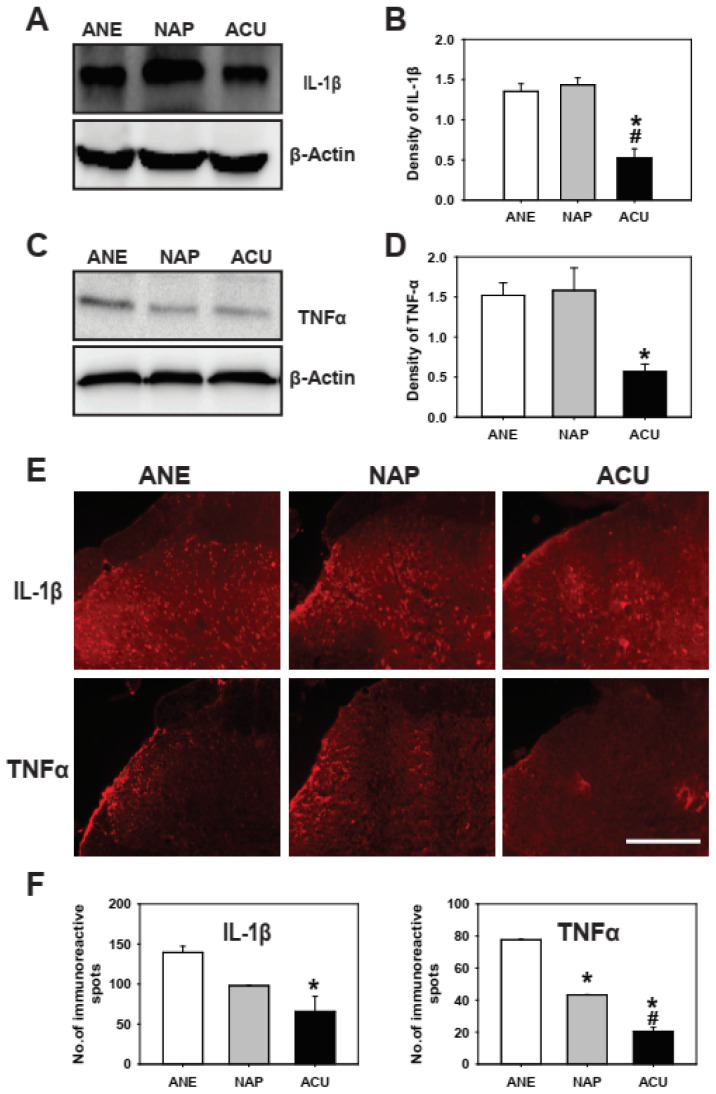
Electroacupuncture (EA) suppresses IL-1β and TNF-α expression in the spinal dorsal horn of rats with neuropathic pain. (**A**,**C**) Representative Western blot images and (**B**,**D**) quantification of IL-1β and TNF-α protein levels in the L4-L6 spinal dorsal horn from the anesthetized control (ANE), non-acupoint stimulation (NAP), and EA (ACU) groups. EA significantly decreased IL-1β and TNF-α expression compared with ANE, and also reduced IL-1β levels compared with NAP. (**E**) Representative immunofluorescence images and (**F**) quantification of IL-1β- and TNF-α-positive cells, showing that EA markedly reduced pro-inflammatory cytokine expression in the dorsal horn. Scale bar = 100 μm. Data are presented as mean ± SEM (*n* = 5 per group for Western blot, *n* = 4 per group for immunofluorescence). * *p* < 0.05 vs. ANE; # *p* < 0.05 vs. NAP.

SNL significantly upregulated pro-inflammatory cytokines in the spinal dorsal horn. Western blot and immunofluorescence analyses revealed that both IL-1β and TNF-α levels were markedly increased in SNL rats compared with naïve controls (Supplementary Figure S4). These findings confirm that SNL robustly induces neuroinflammatory responses that contribute to pain hypersensitivity. We next examined whether EA could suppress this cytokine upregulation. As shown in Figure 4, EA significantly reduced the protein expression of IL-1β and TNF-α compared with both ANE and NAP groups (Figure 4A–D). Consistent with these results, immunofluorescence analysis revealed a marked decrease in the number of IL-1β- and TNF-α-positive cells in the ACU group (Figure 4E,F). Importantly, non-acupoint stimulation failed to produce comparable effects, underscoring the point-specific efficacy of EA.

Together, these results indicate that EA alleviates neuropathic pain, at least in part, by suppressing SNL-induced neuroinflammatory responses through the downregulation of IL-1β and TNF-α expression in the spinal dorsal horn.

### 2.5. Intrathecal CCL2 Reverses the Analgesic Effects of Electroacupuncture

**Figure 5:**
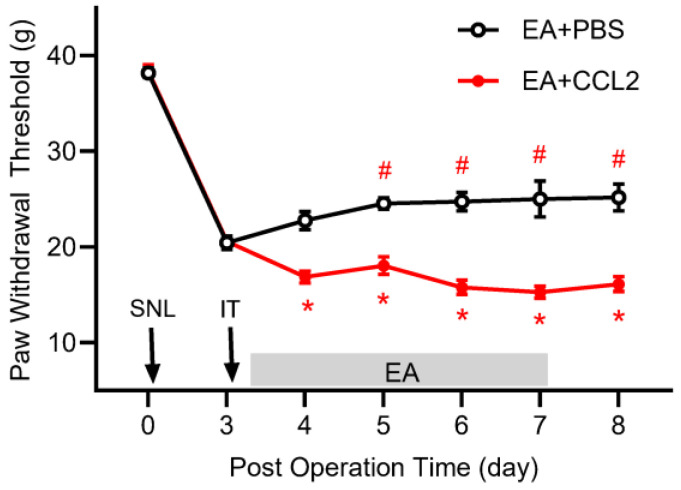
Intrathecal administration of CCL2 abolishes the analgesic effects of electroacupuncture in rats with spinal nerve ligation (SNL). Three days after SNL surgery, CCL2 or PBS was injected intrathecally. Paw withdrawal threshold (PWT) was measured in the both groups. EA significantly increased the PWT in the EA+PBS group compared with pre-EA stimulation baseline (# *p* < 0.05). However, intrathecal CCL2 injection abolished EA’s analgesic effect, as indicated by significantly reduced PWT in the EA+CCL2 group compared with the EA+PBS group (* *p* < 0.05). Data are presented as mean ± SEM (*n* = 8 per group).

To confirm the causal role of the CCL2/CCR2 pathway in EA analgesia, recombinant CCL2 was administered intrathecally. EA significantly increased PWT in the EA+PBS group compared with pre-treatment values. However, intrathecal CCL2 abolished EA’s analgesic effects, as PWT in the EA+CCL2 group was significantly reduced compared to the EA+PBS group (Figure 5). These results strongly support the causal involvement of CCL2/CCR2 signaling in mediating EA’s analgesic effects.

## 3. Discussion

This study provides the first direct causal evidence for the critical role of spinal CCL2/CCR2 signaling in EA-induced analgesia. The complete reversal of EA’s therapeutic effects by exogenous CCL2 administration definitively establishes this pathway as causally necessary rather than merely associated with EA’s mechanism. Our findings demonstrate that EA suppresses spinal CCL2-driven microglial activation to produce its analgesic effects in neuropathic pain. Our results show that in rats with spinal nerve ligation-induced neuropathic pain, point-specific EA treatment at ST36 and GB34 significantly decreased mechanical allodynia and thermal hyperalgesia. This was accompanied by a marked suppression of spinal CCL2 expression and a modulation of microglial CCR2 signaling. Most importantly, intrathecal administration of recombinant CCL2 completely eliminated EA’s analgesic effects, establishing the causal role of CCL2 signaling in EA’s therapeutic mechanism. For the first time, there is concrete proof that CCL2-induced microglial activation is not only associated with but also necessary for the maintenance of neuropathic pain states that EA can successfully reverse. Additionally, we found that EA treatment led to significant decreases in downstream pro-inflammatory cytokines, such as TNF-α and IL-1β, suggesting that CCL2 suppression initiates a larger anti-inflammatory cascade that helps to provide long-lasting pain relief.

**Figure 6:**
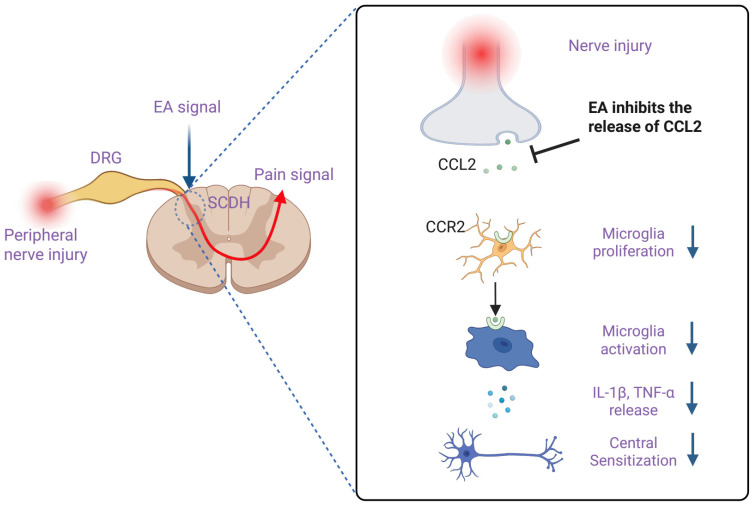
Proposed mechanism of electroacupuncture (EA)-induced analgesia through suppression of spinal CCL2/CCR2 signaling. Peripheral nerve injury induces the release of CCL2 from primary afferent terminals in the spinal dorsal horn (SCDH). CCL2 activates CCR2 on microglia, leading to microglial proliferation and activation, increased secretion of pro-inflammatory cytokines (IL-1β and TNF-α), and central sensitization. EA stimulation at ST36 and GB34 inhibits the upregulation of CCL2, thereby suppressing CCR2-mediated microglial activation and reducing downstream inflammatory cytokine release, ultimately alleviating neuropathic pain.

The many interrelated pathways that affect both the central and peripheral pain processing systems are probably involved in the mechanisms by which EA inhibits the expression of spinal CCL2. The rostral ventromedial medulla and periaqueductal gray are two brainstem nuclei that can directly inhibit spinal microglial activation through noradrenergic and serotonergic projections. By activating descending inhibitory pathways [18,19], EA stimulation at particular acupoints may suppress CCL2 release. As these neuromodulators have been demonstrated to directly reduce inflammation in glial cells, EA-induced release of endogenous opioids and adenosine at the peripheral and spinal levels [27,28] may also aid in CCL2 suppression. EA’s effects on CCL2/CCR2 signaling appear to involve both local mechanisms at the acupoint level and central mechanisms within the spinal cord, as evidenced by the point specificity found in our study, where EA at acupoints ST36 and GB34 was more effective than non-acupoint stimulation. By acting as a local anti-inflammatory, peripheral EA stimulation may lessen primary afferent input, which in turn may lessen the neuronal hyperactivity that produces CCL2 in microglia. In addition to merely deactivating microglia, inhibiting CCL2/CCR2 signaling has a cascading effect on the maintenance of chronic pain states by coordinating a complex inflammatory network that includes astrocytes, infiltrating immune cells, and synaptic plasticity mechanisms. The proposed mechanism is summarized in Figure 6.

Previous studies showing that EA can alter neuroinflammatory responses and microglial function in different pain models provide strong support for our findings. Several studies have reported that EA treatment reduces microglial activation markers including Iba-1 and CD11b in the spinal cord of animals with neuropathic pain, and modulates glial cell morphology and inflammatory responses [20,21,29] consistent with our observations of modulated microglial CCR2 expression. It is well known that CCL2/CCR2 signaling plays a crucial part in the pathophysiology of neuropathic pain, as demonstrated by Zhang et al. [9]. Both resident and bone marrow-derived microglia expressing CCR2 are crucial for pain development, as demonstrated by showing that CCR2 knockout mice display markedly decreased neuropathic pain behaviors. Additionally, research has demonstrated that the production of CCL2 by neurons and astrocytes in the spinal cord leads to central sensitization by means of microglial recruitment and activation, corroborating our finding that the effects of EA on CCL2 result in more extensive anti-inflammatory reactions [10,11]. Electroacupuncture treatment lowers serum and tissue levels of pro-inflammatory cytokines while increasing anti-inflammatory mediators, according to clinical and preclinical research, supporting the anti-inflammatory mechanisms of EA [30,31]. EA’s therapeutic actions may primarily target neuroinflammation, as recent studies have shown that its analgesic effects can be amplified when paired with anti-inflammatory therapies [32].

Despite strong evidence for CCL2/CCR2 involvement in EA analgesia, some studies have emphasized alternative mechanisms that might not directly involve this particular chemokine pathway. With naloxone-reversible effects indicating that μ-opioid receptor activation may be the main mechanism in some experimental conditions, several studies have emphasized the predominant role of endogenous opioid systems in EA-induced analgesia [33]. Adenosine A1 receptor signaling, GABA-mediated inhibition, or modulation of other chemokine pathways, like CX3CL1/CX3CR1, have been the main mechanisms of EA analgesia in other studies, with little attention paid to CCL2 involvement [27,34,35]. Furthermore, depending on the particular pain model being used, some studies have reported varying effects of EA on microglial activation; stronger anti-inflammatory effects were seen in inflammatory pain models as opposed to neuropathic pain models [36]. Conflicts in the literature can result from a number of things, such as different animal species and strains, variations in EA parameters like frequency, intensity, and duration of stimulation, the timing of outcome assessments in relation to treatment, and the particular neuropathic pain models used. Variable sensitivity to EA treatment may arise from varying degrees of CCL2-dependent neuroinflammation caused by the variety of experimental conditions, which range from models of peripheral nerve injury to central pain conditions. While CCL2/CCR2 signaling represents a critical pathway, EA likely involves multiple complementary mechanisms including opioid, adenosine, and GABAergic systems that collectively contribute to analgesic efficacy.

By establishing a causal link between CCL2/CCR2 signaling modulation and EA-induced analgesia in neuropathic pain, going beyond the correlative findings that have defined earlier studies, this work addresses a significant gap in the literature. Even though prior research showed that EA treatment was associated with decreased microglial activation and neuroinflammation, it was unclear how exactly each chemokine pathway contributed to EA’s therapeutic effects. Our proof that the analgesic effects of EA can be reversed by exogenous CCL2 offers vital mechanistic understanding with significant clinical ramifications for improving acupuncture-based pain management techniques. With the help of this study’s mechanistic insights, combination therapies that combine EA with pharmacological CCL2/CCR2 antagonists may be developed, potentially improving treatment outcomes for patients with refractory neuropathic pain conditions while lowering medication side effects and producing synergistic analgesic effects.

The study’s strength lies in its comprehensive experimental design that combines behavioral, molecular, and pharmacological methods to prove causality rather than merely a correlation between CCL2/CCR2 signaling and EA analgesia. Our findings’ translational relevance is increased by the application of point-specific EA stimulation at clinically significant acupoints, and the pathway’s causal significance is strongly validated mechanistically by intrathecal CCL2 administration. Incorporating pain behavioral assessments and multiple molecular markers enhances the validity of our findings and offers a comprehensive description of how EA affects the CCL2-microglial axis.

However, it is important to recognize a few limitations. Since long-term follow-up data is essential for comprehending EA’s therapeutic durability in clinical settings, the study does not assess whether CCL2 suppression and related analgesic effects last past the immediate post-treatment period. We also did not use genetic knockdown techniques or selective CCR2 antagonists to confirm the CCL2/CCR2 mechanism, which might have given us more evidence to support our findings. While our recombinant CCL2 reversal experiment provides strong causal evidence, future studies employing selective CCR2 antagonists or genetic knockdown approaches would provide additional mechanistic validation of this pathway. Additionally, while our non-acupoint control site was selected based on validated criteria, future investigations could benefit from additional control groups including surface electrical stimulation without needle penetration to further exclude potential confounding effects of mechanical stimulation. The exclusive use of male rats represents a significant limitation of this study. Sex differences in pain processing, neuroinflammation, and microglial responses are well-documented, with female animals often showing different inflammatory profiles and analgesic responses to various treatments. Future studies should include both sexes to validate these CCL2/CCR2 mechanisms across genders and assess potential sex-specific therapeutic responses to EA treatment. Additionally, the clinical applicability of these findings from animal models to human neuropathic pain conditions is still unclear due to species-specific variations in immune responses, acupuncture sensitivity, and pain processing mechanisms. To overcome these constraints, future research should include longer observation times, sex-based analyses, more pharmacological treatments, and, in the end, meticulously planned clinical trials to confirm these mechanisms in human participants.

In summary, this study provides the first direct causal evidence for the critical role of spinal CCL2/CCR2 signaling in EA-induced analgesia. The complete reversal of EA’s analgesic effects by exogenous CCL2 administration advances our understanding from correlative observations to mechanistic certainty, conclusively establishing this chemokine pathway as necessary for EA’s pain-relieving effects.

## 4. Materials and Methods

### 4.1. Experimental Animals

Forty-three male Sprague–Dawley rats (200–250 g) were used. Animals were housed in groups of three under a reversed 12 h light/dark cycle with ad libitum access to food and water. All experimental procedures conformed to the Animals (Scientific Procedures) Act (Korea, 2008) and the National Institutes of Health Guide for the Care and Use of Laboratory Animals and were approved by the Institutional Animal Care and Use Committee of Pusan National University (PNU-2019-2370). Every effort was made to minimize the number of animals used and their suffering. Male rats were selected to maintain consistency with established CCL2/CCR2 neuropathic pain literature and to eliminate potential confounding effects of estrous cycle variations on microglial responses and pain behavior.

### 4.2. Experimental Groups

For the main experiment, twenty-seven rats were randomly assigned to one of three groups: (1) anesthetized control without EA (ANE, *n* = 9), (2) non-acupoint EA (NAP, *n* = 9), or (3) acupoint EA at ST36 and GB34 (ACU, *n* = 9). Baseline behavioral assessments were performed on day 0, followed by pre-treatment measurements on day 3. EA was administered once daily two hours after the behavioral tests from days 3 to 7. Behavioral testing was conducted before each EA session. On day 8, L4–L6 spinal cord segments were collected for Western blot and immunofluorescence analyses.

### 4.3. Neuropathic Surgery

Neuropathic pain was induced by L5 spinal nerve ligation (SNL) as previously described [37] with minor modifications. Rats were anesthetized with isoflurane (3% induction, 1.5% maintenance) in a mixed N_2_O chamber. The left L5 spinal nerve was carefully isolated from the adjacent L4 spinal nerve and tightly ligated using 6-0 silk suture. The incision was disinfected with povidone–iodine and closed with wound clips. Animals were returned to their cages for recovery.

### 4.4. Behavioral Assessments

Mechanical allodynia: Mechanical sensitivity was assessed using a Dynamic Plantar Aesthesiometer (Ugo Basile, Varese, Italy). Rats were placed in individual compartments on a wire mesh floor. A calibrated force (0–40 g) was applied to the plantar surface of the affected hind paw using a von Frey filament, and paw withdrawal threshold (PWT) was automatically recorded.

Thermal hyperalgesia: Thermal sensitivity was evaluated using the Hargreaves method [38] with a plantar test apparatus (Ugo Basile). Rats were placed in acrylic chambers on a glass floor, and a focused beam of radiant heat was applied to the plantar surface of the affected hind paw. The cut-off latency was set at 20 s. Paw withdrawal latency (PWL) was defined as the average of three measurements taken at 5 min intervals. Rats were trained for three days prior to testing to reduce trial-to-trial variability (<0.5 s).

Testing sequence was standardized to minimize cross-interference between assessments. Mechanical allodynia testing was performed first, followed by a 30 min recovery interval before thermal hyperalgesia assessment. This sequence was selected because mechanical stimulation produces less sensitization than thermal stimulation, and the 30 min interval allows recovery from any acute sensitization effects. All animals were allowed to acclimate to the testing environment for 15 min before each assessment.

### 4.5. Electroacupuncture Treatment

**Figure 7:**
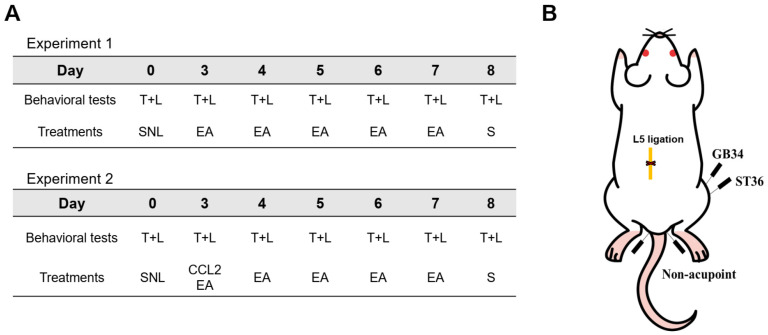
Experimental timeline and acupoint locations. (**A**) Schematic timeline of experimental procedures. In Experiment 1, rats underwent L5 spinal nerve ligation (SNL) on day 0 and received electroacupuncture (EA) at ST36 and GB34 once daily from postoperative day 3 to 7. Behavioral assessments of thermal hyperalgesia (T) and mechanical allodynia (L) were conducted on days 0, 3–8. On day 8, L4–L6 spinal cord segments (S) were collected for molecular analyses. In Experiment 2, rats received intrathecal CCL2 injection on day 3 following SNL, in addition to EA treatment from day 3 to 7. Behavioral assessments and tissue collection were performed as in Experiment 1. (**B**) Diagram of acupoint locations and surgical site. EA was applied at ST36 and GB34. A non-acupoint site near the base of the tail was used as a control. The site of L5 spinal nerve ligation is indicated.

Electroacupuncture (EA) was performed as described previously [16]. Briefly, EA was delivered for 30 min at 2 Hz and 1 mA (pulse width: 1 ms) under isoflurane anesthesia (3% induction, 1.5% maintenance). Stainless steel acupuncture needles (0.25 × 40 mm) were inserted to a depth of 5 mm at ST36 (Joksamli) and GB34 (Yangneungcheon) contralateral to the ligation site. Stimulation was applied using a Pulse Master Multi-Channel Stimulator SYS-A300 (World Precision Instruments, Berlin, Germany). ST36 is located 5 mm below the capitulum fibulae and posterolateral to the knee joint, whereas GB34 is positioned 5 mm superolateral to ST36. In the NAP group, needles were inserted 5 mm lateral to the root of the tail for non-acupoint stimulation. This location was selected based on its anatomical distance from known meridian pathways and different segmental innervation compared to the target area. Experimental timeline and locations of acupoints are presented in Figure 7.

### 4.6. Intrathecal Injection

Direct transcutaneous intrathecal injection was performed using a modified method of Mestre et al. [39]. Sixteen rats were divided into two groups (*n* = 8 each): EA plus PBS injection (EA+PBS) and EA plus CCL2 injection (EA+CCL2). On day 3 post-SNL after behavioral testing, CCL2 (10 μL, 300 ng/mL; R&D Systems, Minnneapolis, MN, USA) or PBS (10 μL) was injected intrathecally via lumbar puncture (L4–L5) using a 25-gauge needle attached to a Hamilton syringe. Correct needle placement was confirmed by observation of tail flick response [39]. EA was administered daily from days 3 to 7 in the experiment.

### 4.7. Western Blot

Five rats from ANE, NAP and ACU groups were deeply anesthetized with pentobarbital and euthanized. L4–L6 spinal cord segments were collected, homogenized in lysis buffer, and centrifuged. Protein concentrations were determined using a Bradford assay. Equal amounts of protein (40 μg) were separated by SDS-PAGE, transferred to nitrocellulose membranes, and blocked with 5% bovine serum albumin (BSA) in TBST. Membranes were incubated overnight at 4 °C with primary antibodies against CCL2 (ThermoFischer, Waltham, MA, USA, #PA534505, 1:500), IL-1β (Abcam, Cambridge, UK, #ab9787, 1:2500), TNF-α (Abcam, Cambridge, UK, #ab9755, 1:2500), and CCR2 (ThermFischer, Waltham, MA, USA, #PA523042, 1:1000). After washing, membranes were incubated with HRP-conjugated secondary antibody (1:2000, Abcam) for 1.5 h at room temperature. Bands were visualized using a CCD imaging system (ImageQuant LAS 4000, Fujifilm, Tokyo, Japan) and quantified with ImageJ software 1.52a (NIH, Bethesda, MD, USA).

### 4.8. Immunofluorescence

Four rats from ANE, NAP and ACU groups were anesthetized and euthanized. L4–L6 spinal cord segments were collected, cryosectioned (20 µm sections, CM3050S, Leica Biosystems, Wetzlar, Germany), and washed with PBS. Sections were blocked for 15 min with CAS Block (Invitrogen, Waltham, MA, USA) and incubated overnight at 4 °C with the following primary antibodies: CCL2 (ThermoFischer, #PA534505, 1:50), IL-1β (Abcam, #ab9787, 1:200), TNF-α (1:100, Abcam), CCR2 (Abcam, #ab9755, 1:50), and Iba-1 (Thermo Fisher, #MA5 27726, 1:500). After washing, sections were incubated for 2 h at room temperature with Alexa Fluor 594 goat anti-rabbit IgG (1:500, Abcam) or Alexa Fluor 488 goat anti-mouse IgG (1:500, Abcam). Sections were mounted with Vectashield (Vector Laboratories, Newark, CA, USA). Fluorescence images were acquired using a microscope (Image MI, Zeiss, Oberkochen, Germany) and analyzed with I-Solution software 11.0 (IMT, Daejeon, Republic of Korea). To control for batch effects, all experimental groups were processed simultaneously using identical reagent lots. Imaging parameters were standardized across all sections, and image acquisition was performed in a single session with the analyst blinded to group assignments.

### 4.9. Statistical Analysis

Sample sizes were determined using a priori power analysis for three-group comparisons (α = 0.05, power = 0.8). Based on preliminary data showing a meaningful difference of 4.0 and standard deviation of 2.5 (Cohen’s d = 1.6), the analysis indicated that *n* = 9 animals per group would be adequate for EA behavioral studies and *n* = 8 animals per group would be adequate for intrathecal experiments. Molecular analyses used *n* = 4–5 per group based on established protocols for Western blot and immunofluorescence studies. Data were analyzed with GraphPad Prism 10.5.0 for Windows (GraphPad Software, Boston, MA, USA). All data are presented as mean ± SEM. Behavioral data were analyzed using two-way ANOVA followed by Duncan’s multiple range test. Western blot and immunofluorescence data were analyzed using one-way ANOVA with Duncan’s post hoc test. Statistical significance was set at *p* < 0.05.

## Data Availability

The data that support the findings of this study are available from the corresponding author upon reasonable request.

## Supplementary Materials

The following supporting information can be downloaded at https://www.mdpi.com/article/10.3390/ijms26189049/s1.
