# Supplementary material for: Electroacupuncture Alleviates Neuropathic Pain by Inhibiting Spinal CCL2-Driven Microglial Activation
Source: Int J Mol Sci. 2025 Sep 17;26(18):9049. doi: 10.3390/ijms26189049 (PMC12470026; doi:10.3390/ijms26189049)
Supplement: Supplementary file 1 [file ijms-26-09049-s001.zip › ijms-3844712-supplementary.pdf]

Supplementary Materials and Methods

S.1. Experimental animals

Twelve male Sprague–Dawley rats (200-250 g) were used. Animals were housed in groups of three under a reversed 12-hour light/dark cycle with ad libitum access to food and water. All experimental procedures conformed to the Animals (Scientific Procedures) Act (Korea, 2008) and the National Institutes of Health Guide for the Care and Use of Laboratory Animals and were approved by the Institutional Animal Care and Use Committee of Pusan National University (PNU-2019-2370). Every effort was made to minimize the number of animals used and their suffering.

S.2. Experimental groups

Twelve rats were randomly assigned to naïve (Naïve, n=6) and spinal nerve ligation group (SNL, n=6). Baseline behavioral assessments were performed on day 0. Following these assessments, six rats were randomly selected to perform spinal nerve ligation to induce neuropathic pain. Subsequent, behavioral assessments were conducted from day 3 to day 8. On day 8, L4–L6 spinal cord segments were collected for Western blot and immunofluorescence analyses.

S.3. Western blot

Three rats from Naïve and SNL group were deeply anesthetized with pentobarbital and euthanized. L4–L6 spinal cord segments were collected, homogenized in lysis buffer, and centrifuged. Protein concentrations were determined using a Bradford assay. Equal amounts of protein (40 μg) were separated by SDS-PAGE, transferred to nitrocellulose membranes, and blocked with 5% bovine serum albumin (BSA) in TBST. Membranes were incubated overnight at 4°C with primary antibodies against CCL2 (ThermoFischer, #PA534505, 1:500), IL-1β (Abcam, #ab9787, 1:2500), TNF-α (Abcam, #ab9755, 1:2500), and CCR2 (ThermFischer, #PA523042, 1:1000). After washing, membranes were incubated with HRP-conjugated secondary antibody (1:2000, Abcam) for 1.5 h at room temperature. Bands were visualized using a CCD imaging system (ImageQuant LAS 4000, Fujifilm, Tokyo, Japan) and quantified with ImageJ software (NIH, Bethesda, MD, USA).

S.4. Immunofluorescence

Three rats from Naïve and SNL group were anesthetized and euthanized. L4–L6 spinal cord segments were collected, cryosectioned (20 μm sections, CM3050S, Leica Biosystems, Wetzlar, Germany), and washed with PBS. Sections were blocked for 15 min with CAS Block (Invitrogen) and incubated overnight at 4°C with the following primary

antibodies: CCL2 (ThermoFischer, #PA534505, 1:50), IL-1β (Abcam, #ab9787, 1:200), TNF-α (1:100, Abcam), CCR2 (Abcam, #ab9755, 1:50), and Iba-1 (Thermo Fisher, #MA5 27726, 1:500). After washing, sections were incubated for 2 h at room temperature with Alexa Fluor 594 goat anti-rabbit IgG (1:500, Abcam) or Alexa Fluor 488 goat anti-mouse IgG (1:500, Abcam). Sections were mounted with Vectashield. Fluorescence images were acquired using a microscope (Image MI, Zeiss, Oberkochen, Germany) and analyzed with I-Solution software (IMT, Daejeon, Korea).

S.5. Statistical analysis

Data were analyzed with GraphPad Prism 10.5.0 for Windows (GraphPad Software, Boston, MA, USA). All data are presented as mean ± SEM. Western blot and immunofluorescence data were analyzed using one-way ANOVA with Tukey post hoc test. Statistical significance was set at $p < 0.05$.

Supplementary figures

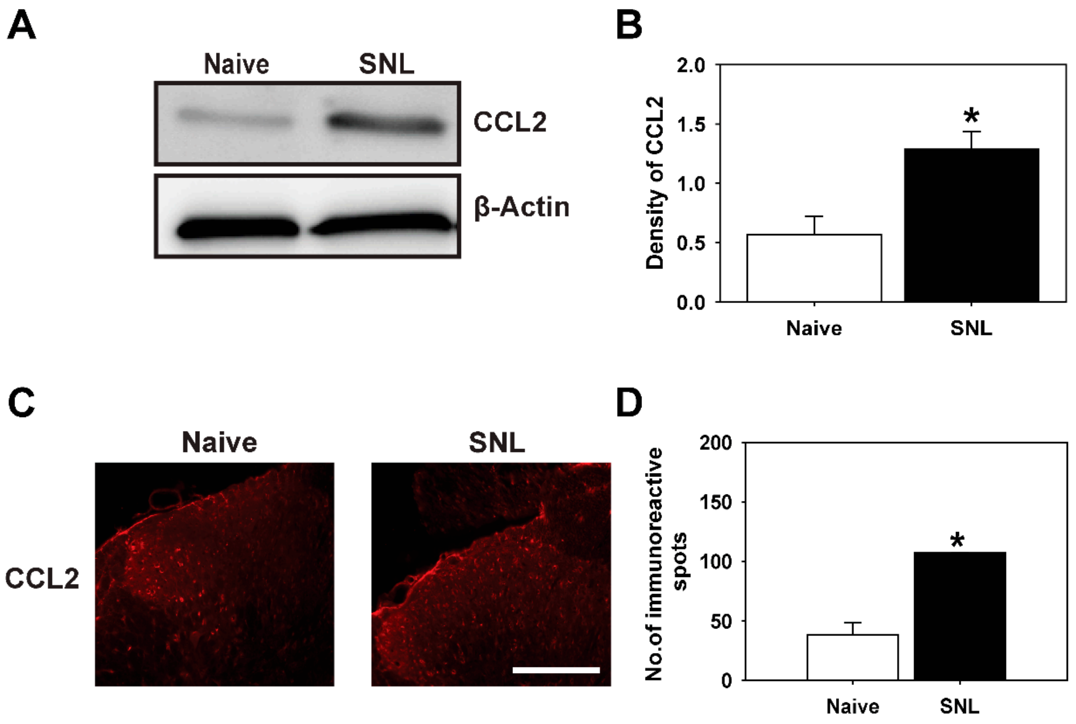

Supplementary Figure S1. Spinal nerve ligation (SNL) increases CCL2 expression in the spinal dorsal horn of rats. (A) Representative Western blot and (B) quantification showing increased CCL2 protein levels in SNL rats compared with naïve controls. (C) Representative immunofluorescence images and (D) quantification of CCL2-positive cells confirming significant upregulation after SNL. Scale bar = 100 μm. Data are presented as mean ± SEM (n=3 per group). *$p < 0.05$ vs. Naïve.

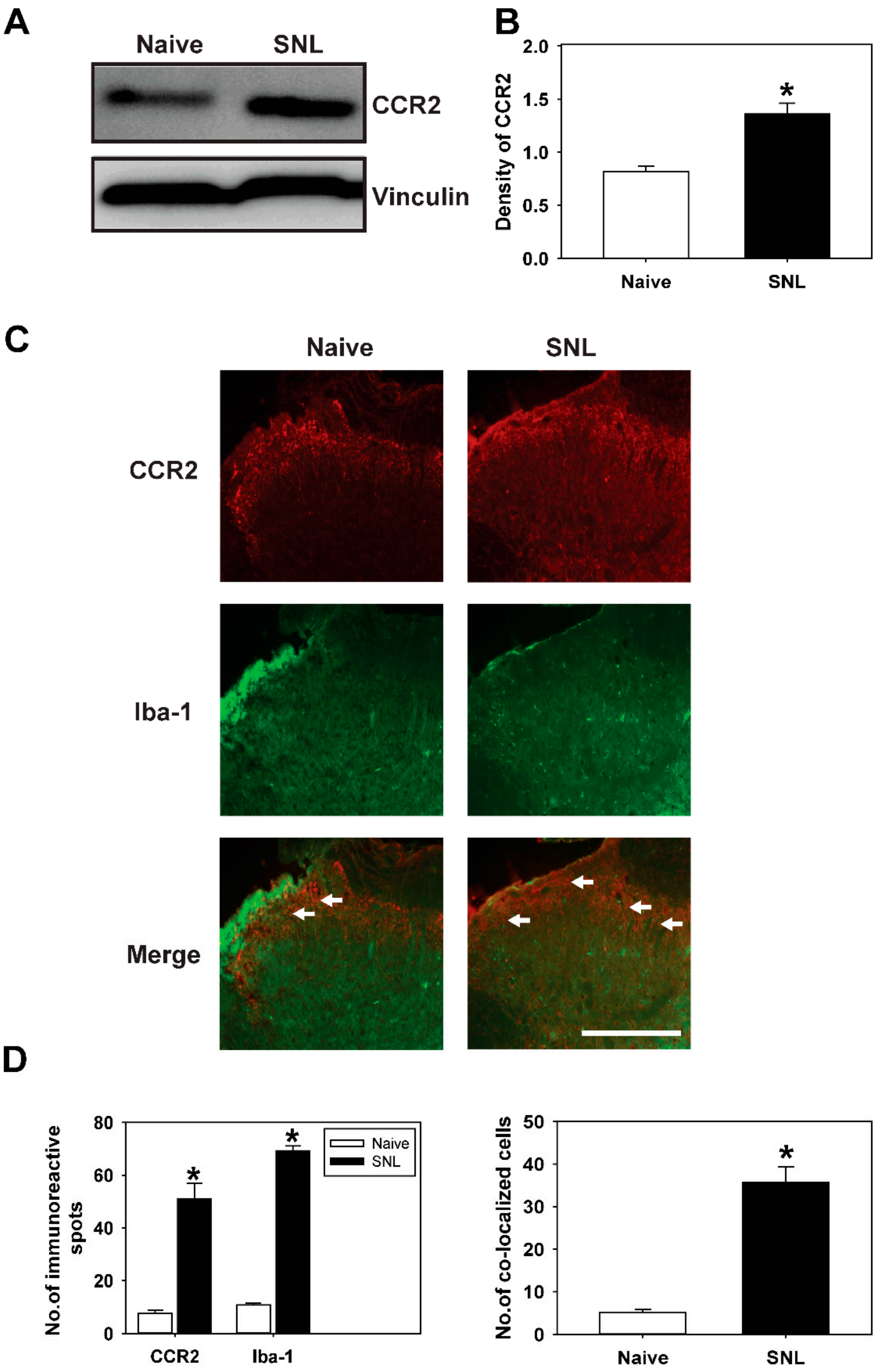

Supplementary Figure S2. Spinal nerve ligation (SNL) increases CCR2 expression and microglial co-localization in the spinal dorsal horn. (A) Representative Western blot and (B) quantification showing elevated CCR2 levels in SNL rats compared with naïve controls. (C) Representative immunofluorescence images showing CCR2 (red) and Iba-1 (green) and (D) quantification demonstrating increased CCR2- and Iba-1–positive cells as well as CCR2/Iba-1 co-localized cells after SNL. Scale bar = 100 μm. Data are presented as mean ± SEM (n=3 per group). *$p < 0.05$ vs. Naive.

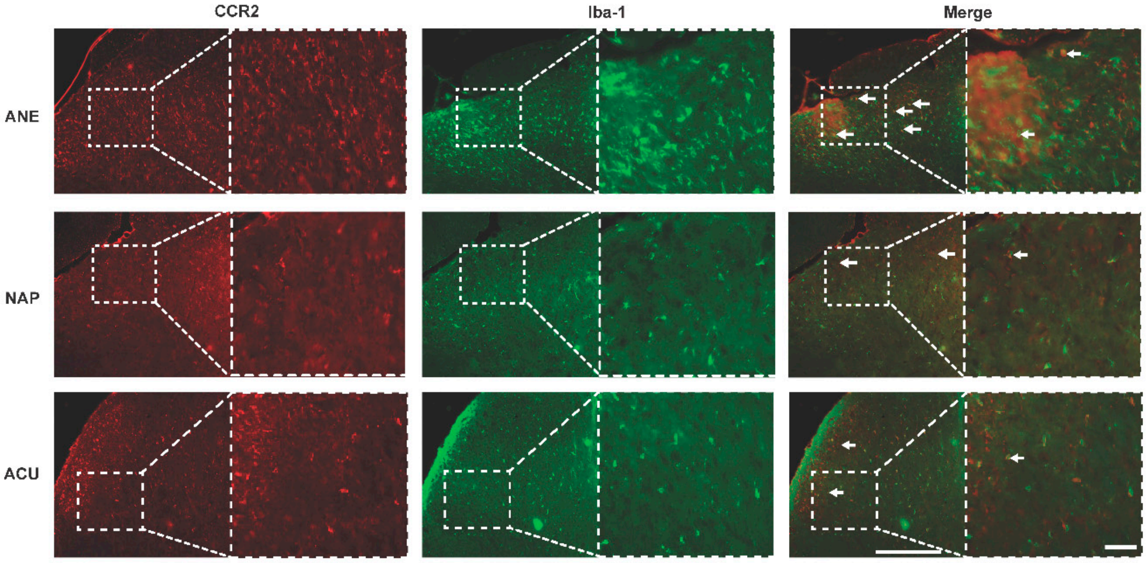

Supplementary Figure S3. High-magnification images confirm EA suppression of CCR2/Iba-1 co-localization. Representative high-magnification immunofluorescence images of CCR2 (red) and Iba-1 (green) in the spinal dorsal horn from ANE, NAP, and ACU groups. Co-localized CCR2/Iba-1 signals (arrows) were markedly increased in ANE and NAP groups but substantially reduced after EA. These images corroborate the quantitative results in Figure 3. Scale bar = 100 μm.

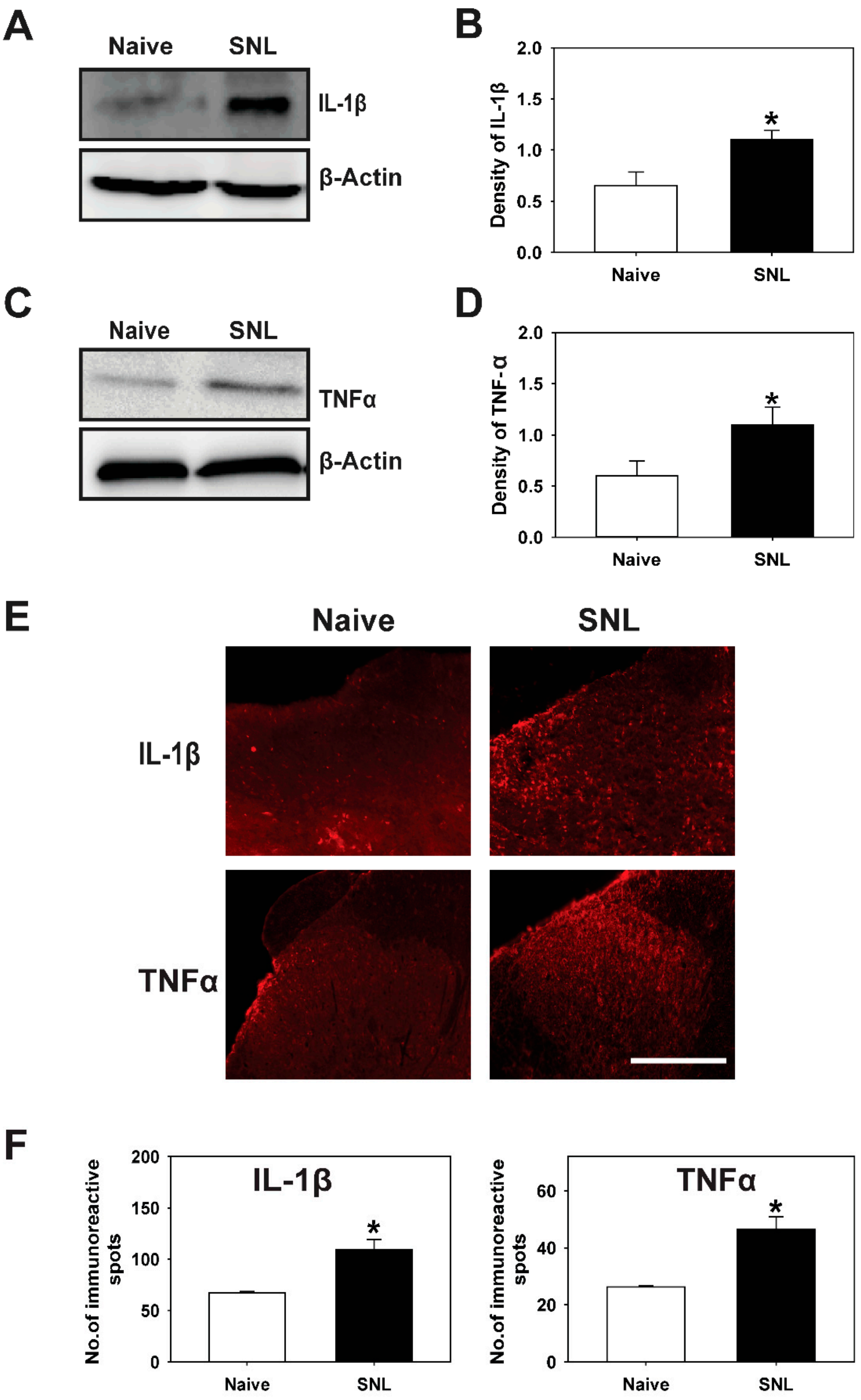

Supplementary Figure S4. Spinal nerve ligation (SNL) increases IL-1β and TNF-*α* expression in the spinal dorsal horn of rats with neuropathic pain. (A, C) Representative Western blot images and (B, D) quantification of IL-1β and TNF-*α* protein levels in SNL rats compared with naïve controls. SNL significantly increases IL-1β and TNF-*α* expression compared with Naive. (E) Representative immunofluorescence images and (F, G) quantification of IL-1β- and TNF-*α*-positive cells showing that SNL markedly reduced pro-inflammatory cytokine expression. Scale bar = 100 μm. Data are presented as mean ± SEM (n = 3 per group). *$p < 0.05$ vs. Naïve.
